# Coping with the Double Crisis: Lake Chilwa Recession and the Great Depression on Chisi Island in Colonial Malawi, 1930–1935

**DOI:** 10.1007/s10745-016-9882-1

**Published:** 2017-01-18

**Authors:** Joseph Nagoli, Erik Green, Wapulumuka Mulwafu, Linley Chiwona-Karltun

**Affiliations:** 1 Swedish University of Agricultural Sciences (SLU), Department of Urban and Rural Development, SE-750 07 Uppsala, Sweden; 2 WorldFish, P.O Box 229, Zomba, Malawi; 3 Lund University, Department of Economic History, 22007 Lund, Sweden; 4 University of Malawi, Chancellor College, P.O. Box 280, Zomba, Malawi

**Keywords:** Lake Chilwa Basin, Malawi, Resource scarcity, Colonial introduced cash crops

Environmental history is to a large extent framed by the neo-classical principle of generalized scarcity that recognizes that nature changes, both independently and in response to human actions, and thus changes the context in which human history unfolds (McNeill 2003). At the same time, political ecology puts more emphasis on power and inequalities as direct drivers of scarcity (Blaikie and Brookfield 1987; Peet and Watts 2004; Robbins 2012). However, both approaches can enhance our understanding of the influences acting upon coping strategies during resource scarcity.

During the 1930s, people in the Lake Chilwa Basin in Malawi had to cope with both the drying up of Lake Chilwa and the global economic depression. We chose to describe this confluence on Chisi Island as the ‘double crisis,’ and it may at first glance seem obvious, but on examination becomes quite complex. In the case of the Lake Chilwa, the colonial administration introduced cotton production on the dry lake bed to boost the economy of Nyasaland in the face of the economic depression. However, the people of Chisi Island successfully resisted cotton farming. The ‘double crisis’ illustrates how power-relations shape scarcity and vice versa.

Environmental scarcity is understood as the decrease in quantity and quality of renewable resources caused by three main processes: i) environmental changes; ii) population increase; and iii) unequal social distribution of resources, also termed ‘structural scarcity’ (de Sherbinin and Dompka 1998). Scarcity therefore becomes a naturally recurring limitation on the availability of resources or goods within an ecological system affecting the organization and functioning of societies. Economic historians, for example, argue that land-labor ratios have had a lasting impact on inequality and power relations because the use of slavery and/or bonded labor has historically been more common in geographical areas of land abundance and labor scarcity (Austin 2008; Green 2014).

Environmental historians address degradation associated with non-renewable resources, environmental change associated with human transformation of renewable resources and environmental rehabilitation, conservation, and preservation (Hughes 2001; Worsted 1993). Similarly, a key feature of African environmental historiography is its emphasis on the conservationist paradigm of managing scarcity of natural resources (Kwashirai 2012; Mulwafu 2010).

Political ecologists have criticized the environmental scarcity framework for neglecting inequality and power relations. Political ecologists emphasize that power and politics determine access to and distribution of specific resources and thus have the potential to create scarcity for the society at large (Blaikie and Brookfield 1987; Peet and Watts 2004; Robbins 2012).

People and organizations obviously control and use different coping strategies during resource scarcities. The differences in coping strategies may or may not be predominantly influenced by unequal power relations (Walker 2006; Vayda and Walters 1999; Zimmerer and Bassett 2003). The relationship between coping strategies and scarcity is multi-causal (Fig. 1). While the interests of powerful actors will often result in limited resources for other users, natural resource scarcity will influence the type of coping strategies employed. Choosing a particular strategy is dependent on ecological history, i.e., past scarcity episodes that enhanced ecological knowledge may structure reactions to new scarcities. However, the linkages among coping strategies and resource scarcity and actors’ historical knowledge raise two questions: what types of natural resources are important for different groups at different times? And, how do different actors gain access to and control over such resources during times of scarcity?

**Fig. 1:**
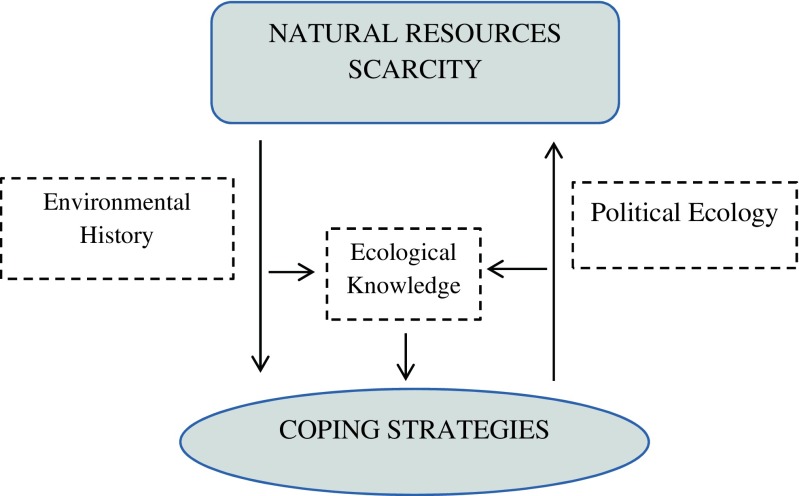
Conceptual framework on the relationship between coping strategies and resource scarcity

Primary records were collected at the Malawi National Archives (MNA) between March and June 2012 in order to get a comprehensive account of the history of Lake Chilwa area. The main sources included Secretariat Records of Nyasaland for the period 1891–1939: administrative annual reports for agriculture, livestock and fisheries; demographic data; climatic data; government policies on agriculture, trade, labor and migration and blue books.^1^

The archival study also provided the basis for primary data collection by identifying key areas of interest. Information from the national archives was used to triangulate data from informants’ oral recollections of major events, historical decisions and organizational players. Archival work was followed by focus group discussions in eight villages on Chisi Island between August and September 2012. The eight villages were purposively selected based on their differences in socio-economic status. Discussions involved fishers, fish processors, traders, transporters, farmers, natural resource governance leaders, and traditional leaders. Men, women and youth formed their own respective groups, which facilitated openness during discussions.

In-depth interviews with key informants on Chisi Island were conducted from August 2012 to March 2013. The interviewees here, 25 elderly residents on the Island and the mainland, were chosen for their knowledge of the lake recessions rather than their representativeness. They had experienced the lake recessions first-hand or were given an account of the recessions of 1967 and 1995 by a first-hand witness. A snowball technique (Bernard 2011) was used to choose informants. These interviews provided a clearer understanding of the complexities in the social structure and the fisheries, as well as the governance of the lake during recessions. In-depth interviews followed consent from the general community and from Traditional Authority (TA) Mkumbira.^2^

The study used an iterative cycle of data collection and analysis, with the intention that the results of the analysis from a particular day guided subsequent collection of data (Walsham 1995; Yin 1994). One fundamental tenet of this approach is that when listening to and documenting narratives from interviews, multiple interpretations, with all their contradictions, are documented simultaneously. These narratives were later grouped in terms of content to understand the effects of the lake recessions on the people of Chisi relative to findings from the National Archives.

## The Lake Chilwa Ecosystem

**Fig. 2:**
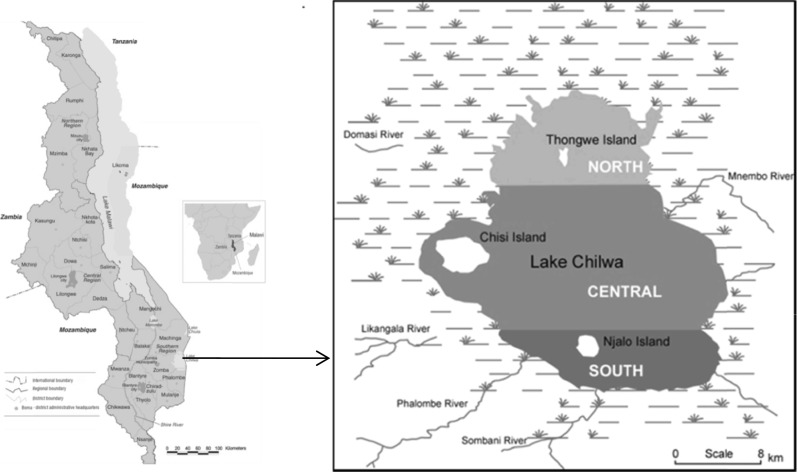
Lake Chilwa and Chisi Island

The Lake Chilwa ecosystem (Fig. 2) is shared between Malawi (3850 km^2^) and Mozambique (1800 km^2^). However, the water level of Lake Chilwa has a long history of fluctuations, mainly due to the balance between rainfall and evaporation during wet and dry seasons (Lancaster 1979; Nicholson 1998). Intermittently, the lake has undergone 12 severe recessions recorded: 1879, 1900, 1914–15, 1922, 1931–32, 1934, 1954, 1960–61, 1967, 1973, 1995, and the most recent in 2012 (Kalk *et al*. 1979; Njaya *et al.* 1996). Three of these were complete dry-ups (1934, 1967, and 1995).

This study was conducted at Chisi Island (formerly known in colonial times as Nchisi). The island is the largest on Lake Chilwa, located between 30^0^ 35′ and 30^0^ 38′ east of Greenwich Meridian and 15^0^ 18′ and 15^0^ 21′ south of the Equator. Chisi Island has an area of roughly 21 km^2^ and is surrounded by marshes to the west and open waters to the east. It was selected for this study on the premise that people’s livelihoods on that island are largely dependent on the lake and would be most affected during lake recessions. Fishing at the island attracts many immigrants that come to fish and trade in both fresh and dried fish.^3^ The population of Chisi Island can therefore swell from 3000 to over 10,000 people between low and peak fishing periods.

### The Great Depression

**Fig. 3:**
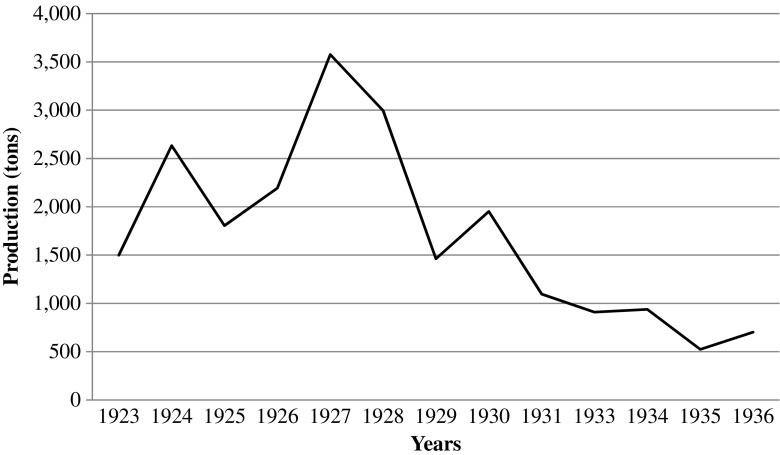
Cotton production in Nyasaland produced by European farmers. 1923–1936. *Source*: Chirwa 1997

The main source of income for the majority of colonial authorities in Africa stemmed from trade, most significantly the export of primary commodities (Leigh 2011). World prices of tropical goods stabilized at a comparatively high rate in the mid-1920s after having fluctuated earlier in the decade. During that period, colonial states in Africa experienced increased revenues, which enabled them not only to finance their recurrent costs, but also to raise development capital from private lenders (Austen 1987). In many British colonies, including Nyasaland, the 1920s marked the beginning of discussions regarding long-term development ambitions, although lack of resources prevented the colonial authorities from formulating serious, coherent development plans. However, the good times were short lived. In the early 1930s, the world economy was hit by the worst global economic recession since the birth of industrial capitalism. Nyasaland, belonging to the periphery of the British Empire and thus only partially integrated in the global capitalist economy, was not directly affected by the deflationary policies that the depression caused. However, the colonial authorities in Nyasaland found it increasingly difficult to raise revenue as the economic depression led to a significant decline in export incomes (Bolt and Green 2015; Madsen 2001). The decline in world market prices and the collapse of European settler agriculture had severe effects on the financial position of the colonial state in Nyasaland. The dramatic decline in European production (Fig. 3) resulted in a significant loss of tax revenues from both domestic and export taxation.

The people of Chisi Island suffered the exogenous effects of the great depression through loss of temporary employment during seasons of low fish catches. With the closure of European commercial farms caused by the Great Depression, islanders’ choices of alternative livelihoods to their seasonal fishing occupation were limited (Mandala 1990; Palmer 1985). Before the depression, European farms in the uplands of Lake Chilwa provided alternative employment to fishing for over 50% of the population of Chisi Island, especially during low fish catch season.^4^ Migration in search of employment on the European farms in southern Rhodesia or in the mines in South Africa was also a limited option due to the decline in demand for labor caused by the global economic crisis. The production of cash crops, such as cotton and tobacco, by Europeans in Nyasaland (now Malawi) had decreased significantly in the early 1930s (Bolt and Green 2015). By the mid-1930s land under tobacco cultivation on European estates had fallen to a mere 20% of the 1927 amount, while cotton production had almost collapsed, decreasing by 1934 to 23% of the 1927 production area (Chirwa 1997) (Fig. 3).

### The 1931–34 Lake Chilwa Water Recession Crisis

In 1931 the levels of Lake Chilwa started to decline due to a long dry spell from March to October. Rains started again in the 1931–32 rainy season and by December flooding occurred only to be followed by three weeks of drought in January 1932. By October 1933, the lake had practically dried up.

In 1932 the islanders and other residents who relied on the lake’s fisheries resources began to suffer from the decline of the Lake Chilwa fish trade, a trend that continued well into 1933 when the fishery completely collapsed. The decline in fish trade forced some traders to start exploring other fishing areas, such as the Upper Shire River at Liwonde, and Lake Malawi in Mangochi. The shift to Liwonde and Mangochi fisheries increased the number of pedal cyclists trading in fish from Fort Johnston (Mangochi) to Zomba markets to a degree that three bicycle-repair shops were established in Zomba District alone. There were also two nationals from Zomba operating a truck to transport fish from Lake Malawi to the urban markets of Zomba, Limbe and Blantyre.^5^

**Fig. 4:**
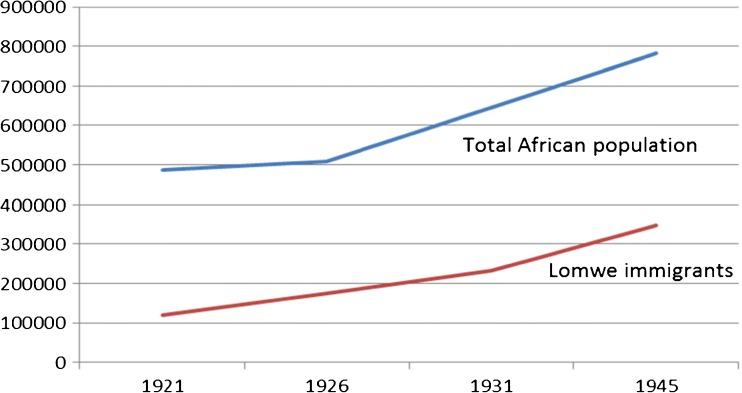
The Lomwe and indigenous population in Shire Highlands and Lower Shire Valley, 1921–1945. *Source:* Chirwa (1997): 542

During the same period from 1920s and 1930s, a huge influx of Yao and Lomwe (Anguru) tribes migrated to Nyasaland from Portuguese East Africa (now Mozambique) fleeing famine and the brutalities of the Portuguese government (Vail 1983). Within Nyasaland, some Lomwe settled in around Lake Chilwa. This rapid immigration (Fig. 4) increased the human population in the Shire Highlands creating intense pressure on land and the region’s natural resources (Chirwa 1997).

The influx of immigrants into the Chilwa ecosystem forced the NAs to order surveillance of all rivers and streams for blockages in order to allow fish to swim upstream for breeding. These measures illustrate not only desperation regarding the loss of fish stocks but also the existence of traditional regulatory mechanisms for protecting and further improving access to scarce resources.

In 1935, the lake started refilling but provided only poor fish catches. Instead, bird hunting became a major source of livelihoods. Bird hunting was said to have reached proportions such that some bird populations began to decline, prompting the state to introduce measures to reduce hunting. For example, a decree was passed by the Zomba District Administrator that listed guinea fowl, whose numbers were believed to have decreased considerably, as protected birds.^6^ In general, Bhima (2006) reported that bird hunting increased by 300–500% in the whole Chilwa wetlands during lake water recessions.

## Cotton Production and Its Resistance on Lake Chilwa and Chisi Island

The lake recession in 1934 had exposed a vast wetland, perceived by the state to be suitable land for cotton production. As attested by the elders from Chisi Island who witnessed (or were told about) the 1934 lake recession during in-depth interviews, the colonial authorities used propaganda to entice NAs to mobilize their subjects for a cotton production scheme on the exposed wetland. The elders claimed that the state believed that the recession was permanent and that the lake was unlikely to refill. This view was contrary to the islanders’ previous experiences of periodic recessions and refills of Lake Chilwa. As one key informant explained:

[...] we were all told by our leaders that the azungu [Colonial Authorities] in Zomba had ordered that we grow cotton on the lake. My father explained that people laughed at the azungu as confused people who did not understand Chilwa [the lake] [...]. (Elder Nyatwa^7^)

The choice of cotton farming on Lake Chilwa was not just to provide an alternative source of income to replace fishing for the islanders, but was also an important crop in the broader scheme of increasing state revenue, especially during the economic depression.^8^ Interviews with elders in the basin indicated that the colonial state pushed for cash crop farming in the entire Lake Chilwa Basin for tax revenues. In the Lake District Report of 1923, the colonial authorities had identified rice, cotton and rubber as potential cash crops to be promoted for payment of taxes by the African farmers in the lakeshore areas that included other inland wetlands. Cotton was given significant emphasis as a peasant crop because it contributed to the imperial plans of enhancing self-sufficiency of metropolitan textile industries that promoted exports and thus provided the colonial authorities with a much needed source of revenue (Mandala 2005).

The campaign for cotton production at Chisi and the wetland was met with resistance.^9^ Elder informants indicated that the people from Chisi Island indirectly refused to cultivate cotton by boiling or roasting the cotton seed before planting. As one elder testified:

[…] people could not directly say no to start growing cotton instead they used several tactics such as roasting or boiling the seed. When the seeds were planted, they couldn’t germinate and the white agricultural extension officers were surprised and got angry. [...] there were few cowards that planted and the seed germinated but these were not many […]. (Elder Kamangeni)

Cotton, a highly labor-intensive crop, was also resisted elsewhere in southern Africa as farmers faced inadequate labor compensation (Brass 1999; Scott 1990). In Mozambique, Isaacman (1996) showed that peasants worked at lowering yields by boiling cotton seeds before planting or by refusing to uproot and burn old plants, sometimes directly refusing to sell cotton or burning already harvested cotton.

## Conclusion

Although it might have been expected that the colonial authorities’ cotton drive would be welcomed by the Chisi Islanders given the lack of income opportunities, they in fact resisted the introduction of cotton. This was because the islanders had been with adequate knowledge of the lake level dynamics. The analysis of a focus group discussion at the Chisi Island indicated that the islanders did not interpret the 1934 water recession as any different from known recessions of the past. Their historical knowledge of the ecosystem and their identification as fishermen for generations provided confidence that the 1934 recession was not permanent. For them, the drying was temporary and could be weathered as long as they had other natural resources, such as birds, that provided food in place of fish.

Aside from ecological knowledge that provided assurance that the lake would return, another issue that emerged during focus group discussions was that of religion. The people of Lake Chilwa feared that transforming the lake ecosystem for agriculture would adversely affect spirits associated with religious shrines. Lake Chilwa has several shrines, especially on Chisi and Chidyamphiri Islands, which accommodate ancestral spirits believed to be important for regulating the lake and its ecosystem services. The existence of these shrines was brought to the attention of the Honorable Chief Secretary by the game warden in Lilongwe in 1930 as reason to designate Chidyamphiri Island as a game reserve for protection of these shrines and the python spirits.^10^ The people of Lake Chilwa have throughout history offered sacrifices during recessions and during times of hunger. While this faith has now lost virtually all its followers, the shrines continue to be of cultural and historic value. John McCracken has described how resistance to the colonial economy was inspired by religious beliefs with the growth of powerful popular nationalism that contained the seeds of a new authoritarianism (McCracken 2012).

The colonialists focused narrowly on the economic consequences of the depression without considering the social and cultural context. Colonial authorities, with little understanding of the physical and social dynamics on the island, introduced a new commodity both as a subsistence strategy for the people and as a potential source of government revenue. The introduction of cotton was not only inconsistent with the historical and cultural roots of the community but, more importantly, it made little sense to the islanders given their knowledge of temporal fluctuations in water levels. The islanders knew that the lake would soon be filled again and therefore resisted the introduction of cotton, a decision when the lake refilled in 1935, and livelihoods based on the fisheries and birds hunting flourished again.

What was striking in the case of Lake Chilwa is that the successful resistance can be explained by the islanders’ ecological knowledge and social cohesion. The unity of people in Chilwa in resisting colonial decisions to turn Lake Chilwa into a cotton farm is impressive considering that there must have been divisions among the individuals with different interests, based on age, political conviction, literacy and class. The unity was not self-evident even in the fairly homogenous and small area of the Lake Chilwa Basin. The 1934 resource scarcity was perceived as a problem of limited temporal access to natural resources rather than a problem of absolute scarcity (Walker 2004). The islanders, who may be perceived as politically weak, collectively possessed the capacity to withstand scarcity through ecological knowledge and unity based on common values, including religion.

The local communities at Chisi Island exercised power through processes that took shape around them, inside and outside of formal institutions. This situation demonstrated that coping with resource scarcity is complex and multifaceted. On one hand is the struggle to satisfy immediate needs and on the other is the long-term perspective of future possibilities.

Lake Chilwa is an important case for examining contemporary challenges faced by different interest groups during natural resource scarcity. Specifically, the conflict between Chisi Islanders and the state explains how decision-making with respect to radical resource and political fluctuations drives the livelihoods of rural communities. The attempted introduction of cotton to the lake, and its resistance, shows how government agricultural policies were shaped by a misunderstanding of the environment. Without the depression and the drying up of the lake there would have been no impetus to promote cotton for raising government revenue. The Chisi Islanders successfully resisted the introduction of cotton because they had a long term understanding of the local ecological system as well as a cultural heritage that unified them. Sometimes a “competing claims approach” that looks at power, the resource itself, culture, and local and national economic pressures is required in understanding the resolution of conflicting interests during resource scarcity.

## Footnotes

1 The colonial ‘Blue Books’ are annual returns of statistical information from British colonies. It originated from a request from the Commons Select Committee of Finance in 1817.

2 A ‘traditional authority’ is a non-elected leader holding office based on his or her ethnic group. The traditional leadership was first included in the colonial administration as part of the indirect rule system formerly known as ‘Native Authorities’ (NA) and is now integrated into the local government structure (Green 2011).

3 Malawi National Archives, Zomba District Administration Report 1919–20 (MNA S1/1128/19)

4 Personal communication Traditional Authority Mkumbira

5 MNA S1/247F/34.

6 Malawi National Archives, Zomba District Administration Report 1935 (MNA S1/79F/36)

7 All the names included for quotes are pseudonyms except for those of chiefs.

8 Malawi National Archives, AGRICULTURE- Lake District Report 1923 by the Assistant Director of Agriculture in 1922 (MNA S1/2433/23)

9 MNA S1/88F/35

10 Malawi National Archives: A letter from the Game Warden in Lilongwe to the Honorable Chief Secretary dated 23/1/1930.
